# Supplementary material for: SyNDI: synchronous network data integration framework
Source: BMC Bioinformatics. 2018 Nov 6;19:403. doi: 10.1186/s12859-018-2426-5 (PMC6219086; doi:10.1186/s12859-018-2426-5)
Supplement: Supplementary file 5 — Files of the example presented in Additional file 4. This additional file is a zip package that contains all files used in Additional file 4. Detailed descriptions of each of these files are presented in a README file included in this zip package. (GZ 30411 kb) [file 12859_2018_2426_MOESM5_ESM.gz › SyNDITools/Meme2Fimo/meme_4.9.0_4.tar.gz!/meme_4.9.0/website/html/motif_discovery.html]

MEME Suite - Submit A Job


|  |  |
| --- | --- |
|  | MEME Suite The MEME Suite web application requires the use of JavaScript   Javascript doesn't seem to be available on your browser.  Documentation on Motif Search Tools  The MEME Suite allows you to discover motifs in groups of related DNA and protein sequences. Click on each of the tools listed below for more information on the different kinds of motif discovery searches you can do.   - MEME   discovers **multiple, ungapped motifs** - GLAM2   discovers **single, gapped motifs** |
